# Supplementary material for: ATP2A2 SINE Insertion in an Irish Terrier with Darier Disease and Associated Infundibular Cyst Formation
Source: Genes (Basel). 2020 Apr 28;11(5):481. doi: 10.3390/genes11050481 (PMC7291265; doi:10.3390/genes11050481)
Supplement: Supplementary file 1 [file genes-11-00481-s001.zip › Figure_S1.pdf]

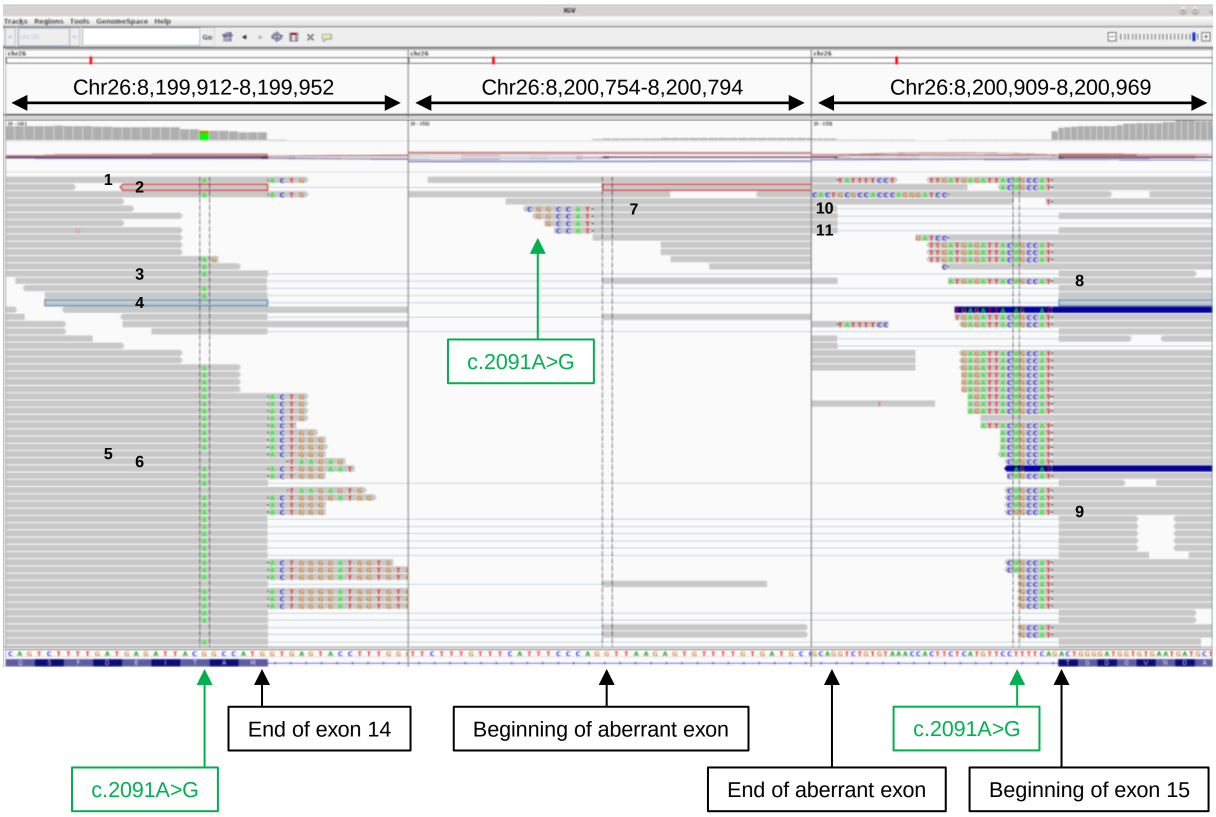

Figure S1. RNA-seq data confirming the aberrant splicing. The figure shows an IGV screenshot of the 50 bp read alignments of RNA-seq data derived from lesional skin of the affected Irish Terrier. Soft-clipped bases not aligning to the genomic reference are shown. Please note that this is a split screen view showing three different genomic locations simultaneously. The genomic coordinates are indicated at the top, the relative positions within the *ATP2A2* gene are indicated at the bottom. The position of the c.2091A>G variant is indicated in all three windows. The reference transcript NM_001003214.1 represents the A-allele, while the CanFam3.1 reference genome assembly represents the G-allele. The allelic ratio between transcripts from the A-allele and the G-allele is 89:21. Numbers from 1 to 11 indicate characteristic reads that allow to infer several splicing events.

1: Read from A-allele, correct splice junction exon 14/15 as indicated by the soft-clipped bases (n = 27)
2: Read from G-allele, splicing of exon 14 to an aberrant exon (n = 7)
3: Read from A-allele, correct splice junction exon 14/15 (n = 19)
4: Read from G-allele, correct splice junction exon 14/15 (n = 2)
5: Read from G-allele, correct splice junction exon 14/15 as indicated by the soft-clipped bases (n = 1)
6: Read from G-allele, splicing of exon 14 to aberrant exon as indicated by the soft-clipped bases (n = 2)
7: Read from G-allele, splicing of exon 14 to aberrant exon as indicated by the soft-clipped bases (n = 2)
8: Read from A-allele, correct splice junction exon 14/15 as indicated by the soft-clipped bases (n = 31)
9: Read from G-allele, correct splice junction exon 14/15 as indicated by the soft-clipped bases (n = 1)
10: Read inferred to originate from G-allele, splice junction from the aberrant exon to exon 16 (n = 2)
11: Read inferred to originate from G-allele, splice junction from the aberrant exon to exon 15 (n = 2)

Similar results were obtained with an independent RNA sample from non-lesional skin of the affected dog (data not shown).
